# Recent Progress in the Diagnosis and Management of Type 2 Diabetes Mellitus in the Era of COVID-19 and Single Cell Multi-Omics Technologies

**DOI:** 10.3390/life12081205

**Published:** 2022-08-08

**Authors:** Krisztina Kupai, Tamás Várkonyi, Szilvia Török, Viktória Gáti, Zsolt Czimmerer, László G. Puskás, Gábor J. Szebeni

**Affiliations:** 1 Department of Physiology, Anatomy and Neuroscience, Faculty of Science and Informatics, University of Szeged, Közép fasor 52, 6726 Szeged, Hungary; 2 Department of Internal Medicine, University of Szeged, Korányi fasor 8, 6720 Szeged, Hungary; 3 Laboratory of Functional Genomics, Biological Research Centre, Temesvári krt. 62, 6726 Szeged, Hungary; 4 Department of Biochemistry and Molecular Biology, Faculty of Medicine, University of Debrecen, Life Science Building, Egyetem tér 1, 4032 Debrecen, Hungary; 5 Avidin Ltd., Alsó kikötő sor 11/D, 6726 Szeged, Hungary; 6 CS-Smartlab Devices Ltd., Ady E. u. 14, 7761 Kozármisleny, Hungary

**Keywords:** type 2 diabetes mellitus, insulin resistance, inflammation, obesity, CVD risk, COVID-19

## Abstract

Type 2 diabetes mellitus (T2DM) is one of the world’s leading causes of death and life-threatening conditions. Therefore, we review the complex vicious circle of causes responsible for T2DM and risk factors such as the western diet, obesity, genetic predisposition, environmental factors, and SARS-CoV-2 infection. The prevalence and economic burden of T2DM on societal and healthcare systems are dissected. Recent progress on the diagnosis and clinical management of T2DM, including both non-pharmacological and latest pharmacological treatment regimens, are summarized. The treatment of T2DM is becoming more complex as new medications are approved. This review is focused on the non-insulin treatments of T2DM to reach optimal therapy beyond glycemic management. We review experimental and clinical findings of SARS-CoV-2 risks that are attributable to T2DM patients. Finally, we shed light on the recent single-cell-based technologies and multi-omics approaches that have reached breakthroughs in the understanding of the pathomechanism of T2DM.

## 1. Introduction

Diabetes Mellitus (DM) is classified into three categories: type 1 diabetes mellitus (T1DM, juvenile diabetes), type 2 diabetes mellitus (T2DM, adult-onset diabetes), and other special types such as gestational diabetes (GDM), endocrinopathies, drugs, and chemical-induced forms, among which T2DM represents nearly 90% of cases (Table 1). The main hallmark of DM is hyperglycemia ≥126 mg/dL (7.0 mmol/L) and a normal fasting blood sugar of between 70 and 99 mg/dL (from 3.9 to 5.5 mmol/L) [1]. Here, we only mention T1DM and GDM and review T2DM; the other forms are described in Table 1 and reviewed elsewhere [2,3,4,5,6,7]. T1DM is characterized by a lack of insulin production with pancreatic β-cell destruction through an idiopathic autoimmune mechanism. GDM is a frequent pregnancy complication in which spontaneous hyperglycemia appears, even in non-obese women, affecting approximately 10–14% of pregnancies worldwide [8]. GDM can be controlled with a low carbohydrate diet and/or insulin administration during pregnancy, and glucose metabolism should be regularly monitored after delivery because it can develop into persistent T2DM and even cardiovascular disease (CVD) in the mother and/or in the descendant [8]. Human placental lactogen is one of the most important factors during the development of gestational diabetes, and it is characterized by low insulin sensitivity or insulin resistance (IR) that leads to chronic hyperglycemia during pregnancy. This hormone is capable of provoking alterations and modifications in the insulin receptors [9]. T2DM also develops because of IR and/or faulty insulin secretion. T2DM is now being diagnosed more often in children and adolescents with obesity due to β-cell malfunction or unresponsiveness to insulin in the organs; in this vicious circle, the insulin secretion is insufficient to compensate for IR [10,11]. IR is linked to environmental factors, low physical activity, high-fat diet, obesity, aging in western society, and genetic background [10,12,13,14]. Genetic predisposition has been described for the development of T2DM via the dysfunction of several genes. Early genome-wide association studies (GWAS) identified approximately 70 genes with mutations or with single-nucleotide polymorphisms (SNPs), and recent multi-ancestry genetic studies found more than 500 risk loci associated with a higher risk for the manifestation of T2DM; the full list of these loci has been reviewed elsewhere [15,16,17]. T2DM represents the disturbance of the metabolomic homeostasis via a low insulin:glucagon ratio, with decreased insulin and increased glucagon production pushing the balance toward hyperglycemia. While insulin supports anabolic processes, the deposition of glucose, the production of proteins, and reductions in free fatty acids, glucagon supports catabolic processes such as the mobilization of glucose and the release of free fatty acids from adipose tissue [18,19]. The conditions of elevated blood glucose and free fatty acid level influences the composition of the microbiota in the gut and the release of pro-inflammatory mediators, and the generation of reactive oxygen species leads to mitochondrial dysfunction and endoplasmic reticulum stress at a sub-cellular level [20,21].

Since hyperglycemia is the most frequent measure and pathologic trait of T2DM, our current report addresses the “Hyperglycemia: From Pathophysiology to Therapeutics” Special Issue in *Life* journal. Our aim is to overview different aspects of T2DM such as prevalence, economic burden, and signs. Non-pharmacological and pharmacological therapeutic interventions such as glucose-lowering efforts are reviewed. Comorbidities of T2DM, as well as the pathophysiology and prognosis of COVID-19 in T2DM, are also discussed. Finally, the recent results of multi-omics technologies are summarized, identifying high-risk factors for T2DM.

## 2. Prevalence of T2DM

DM has become one of the most frequent global health problems (nearly 90% T2DM), with an incidence of 422 million in 2018. The prevalence of DM is continuously increasing and will reach 439 million subjects by 2030, and according to the International Diabetes Federation (IDF), it is estimated to affect 592 million people worldwide in 2035 [22,23]. The emergence of T2DM cases rose from 5.1% to 6.5% of the population in the USA from 1994 to 2002 [24], and T2DM further increased to 8.5% in the US population (91.2% of all diabetic diseases were T2DM) according to a 2016–2017 survey [25]. The fastest economic and technological developments in China contributed to western-type lifestyles and caused a dramatic increase in the prevalence of T2DM, measured at 9.1% in 2016 [22]. In Switzerland in 2012, the prevalence of T2DM was 6.3% (9.1% in men and 3.8% in women) and increased age, obesity, and male gender showed positive correlations in a representative, cross-sectional study with 6181 subjects [26]. The global incidence of T2DM is around 7.2%, and it is expected to reach 9% by 2040 [27].

## 3. Economic Burden of T2DM

Statistics about the expense of diabetes on the economy are mainly available for overall DM, but the economic burden of T2DM corresponds to 90% of DM in proportion with its incidence. DM is the ninth major condition that reduces the life expectancy of men and women by 13.2 and 13.9 years, respectively [28,29]. In 2015, five million deaths caused by DM and its complications were reported, contributing to the loss of active workers and consumers of the global economy [30]. The primary and secondary costs of diagnosed DM were 132 billion USD in the USA in 2002 [24] and increased to 327 billion USD in 2017, and the total costs associated with pre-diabetes (43.4 billion USD), GDM (1.6 billion USD), and undiagnosed DM (31.7 billion USD) were estimated to comprise a 403.9 billion USD economic burden on US society in 2017 [31].

The IDF estimates that the global cost of diabetes was 673 billion USD in 2015, which is projected to rise to 802 billion USD in 2040. More recently, Bommer et al. estimated the global cost burden of treating diabetes to be 1.31 trillion/year USD, an estimate considering both direct costs and production losses due to morbidity or premature mortality [28]. According to the study of Einarson et al., the average healthcare cost for a T2DM patient without CVD is 8310/year USD, while the cost for a T2DM patient with CVD is 15,105/year USD [29]. The trend of global T2DM burden was found to be similar to that of total diabetes (including type 1 and type 2 diabetes mellitus), while the global age-standardized rate of mortality for T1DM has declined [30].

## 4. Signs and Symptoms of T2DM

Symptoms used to diagnose diabetes are as follows: (1) fasting blood glucose ≥126 mg/dL, both (2) the oral glucose tolerance test (OGTT) and (3) random plasma glucose are ≥200 mg/dL, and (4) hemoglobin A1c (HbA1c, glycohemoglobin) ≥6.5% (Table 2) [7]. Further symptoms of diabetes include thirst, polydipsia, polyuria, fatigue, constant hunger, weight loss, dry mouth, and blurred vision [32]. These measures do not discriminate between T1DM or T2DM, and only one parameter is enough to define DM. T2DM is mainly diagnosed with pancreatic β-cell dysfunction and peripheral insulin resistance [7]. These changes lead to decreased glucose uptake (hyperglycemia), diminished peripheral fat uptake (dyslipidemia), compromised amino acid uptake, and higher glucagon production [33]. However, patients with T2DM often show high concentrations of insulin and C-peptide. Autoantibodies—particularly against islet cells, insulin, glutamic acid decarboxylase, and tyrosine phosphatase (islet cell antigen 512)—are not generally detected in patients with T2DM [34]. A high leptin level in the sera is frequently associated with insulin resistance and T2DM [35]. We recently reported an increased leptin level in apolipoprotein-overexpressing (APOB-100) mice fed a high-fat diet [36]. The connection between high serum leptin concentration and CVD has also been reported in T2DM [37]. Obesity, endothelial dysfunction and hypertension have also been reported in T2DM patients with high leptin concentrations [38].

## 5. Non-Pharmacological Treatments of T2DM: Exercise and Diet

It has long been known that sedentary lifestyle and increased calorie intake lead to obesity, which is the major risk factor for developing T2DM [39]. In 2015, almost 2 billion people were affected by obesity worldwide [40], and approximately 2.4 and 2.3 million deaths were caused by high body mass index (BMI)-related diseases including T2DM in women and men, respectively [41]. The Global Burden of Disease Study showed that obesity affected 38% of women, 37% of men, 23% of girls, and 24% of boys in 2013 [42], and it is projected that obesity will increase in 44 countries by 2025 [43]. Early randomized clinical trials that enrolled patients with impaired glucose tolerance led to the conclusion that at least 30 minutes of daily physical activity can reduce the incidence rate of T2DM by 46%–67% depending on the condition of the subject and the type of exercise [44,45,46]. Therefore, preventative dietary management and sporting for high-risk individuals are suggested, and patients with T2DM are first treated with diet modification and suggestions for regular physical exercise [47]. Regular daily physical activity is strongly advised for the prevention of T2DM in high-risk individuals with sedentary lifestyle and obesity [48]. A dose–response relationship between regular exercise and its beneficial metabolic effects is well-accepted [39]. A prospective follow-up study of 32,002 men for 18 years showed that at least 150 min/week weight training or aerobic exercise reduced the risk of T2DM by 34% or 52%, respectively [49]. Umpierre et al. showed that at least 150 minutes of structured exercise per week can reduce the HbA1c level with greater benefits than shorter training [50]. In our study, regular physical exercise (45 minutes of running five times a week) reduced body weight, serum triglyceride levels, and the expression of pro-inflammatory mediator TNF-α in mice fed a high-fat diet as part of an apolipoprotein B-100-overexpressing murine model of obesity [36]. The contribution of higher concentrations of pro-inflammatory cytokines, such as TNF-α, IL-1β, and IL-6, for the development of T2DM and the protective effect of regular exercise was reviewed by Karstoft et al. [51]. Clinical trials have shown that lifestyle changes including physical exercise with diet modification are more effective than pharmaceuticals in preventing T2DM [48]. Dietary recommendations favoring the intake of whole grains, legumes, vegetables, and fruits at the expense of highly refined carbohydrates, sugar-sweetened beverages, refined grains, and red meat may ameliorate T2DM or patient conditions [52,53]. The Mediterranean diet (a low-carbohydrate/high-protein diet) and vegan/vegetarian diets are reported to improve metabolic conditions in T2DM [54]. The protective effect of the Mediterranean diet was shown by Keys based on a follow-up study of 11,579 men for 15 years. Diets with high intakes of saturated fatty acids were associated with CVD, but monounsaturated fatty acids (olive oil) were protective against CVD [55]. The consumption of the Mediterranean menu consisting of fruits (antioxidants), vegetables, red wine (polyphenols), fish, and olive oil (monounsaturated fatty acids) may have anti-inflammatory protective effects against T2DM [56]. A low-carbohydrate diet (<130 g/day) lowers glycemia, HbA1c, and the need for medication [47,57]. A 2-year trial with 322 obese subjects showed that both Mediterranean and low-carbohydrate diets were more effective for weight loss and decreases in C-reactive protein levels compared to a low-fat diet [58]. In a 74-week clinical trial, Bernard et al. showed that a vegan diet reduced the HbA1c level and low-density lipoprotein (LDL)-cholesterol more efficiently than a low-fat diet [59]. Taken together, physical exercise, weight management, daily calorie intake, and the composition of food and beverages should be tailored to each patient’s condition while considering several parameters such as the age of the patient, comorbidities, geographical area (climate), and the suggested pharmaceutical intervention [60].

## 6. Pharmacological Treatment of T2DM

In most cases, initial drug therapy starts with monotherapy based on clinical laboratory parameters such as HbA1c. The five-year VERIFY study in T2DM demonstrated the long-term clinical benefits of early combination treatment with vildagliptin and metformin in comparison to monotherapy [61]. The oral hypoglycemic medications approved by U.S. Food and Drug Administration (FDA) indications are summarized in Table 3. Sulfonylureas (SU) were the first drugs to stimulate insulin secretion, and they have been used since 1954 [62]. Later, non-sulfonylurea secretagogues, i.e., meglitinides, were introduced, e.g., Repaglinide in 1997 [63]. Both SU and meglitinides stimulate insulin secretion by inhibiting ATP-dependent K^+^ channels of pancreatic beta cells [63]. However, meglitinides are not the golden standard in T2DM therapy because its effects last less long than those of SU and can cause hypoglycemia in diabetic patients with chronic kidney disease (CKD) [64].

Nowadays, metformin is among the preferred initial pharmacologic agents for T2DM according to the EASD (European Association for the Study of Diabetes) and ADA (American Diabetes Association) considering comorbidities and lifestyle modifications [67]. The advantages of metformin are its efficacy, weight neutrality, low cost, low-risk of hypoglycemia, and good safety profile with particular cardioprotection [68]. The prescription of metformin should be considered as a monotherapy or in combination with other glucose-lowering drugs for the therapy of T2DM [67]. In cases of renal insufficiency and an eGFR (estimated glomerular filtration rate) of >30 mL/min/1.73 m^2^, early combination therapy can be considered in some patients at treatment initiation to extend the time to treatment failure. In a high-fat diet rat model, metformin combined with a single low dose of streptozotocin-induced diabetes mellitus showed a renoprotective effect following per os administration. Moreover, lipid parameters such as triglyceride (TG), total cholesterol (TC), and LDL-c levels were significantly decreased following metformin treatment, whereas high-density lipoprotein (HDL)-cholesterol was increased. The authors speculate that the underlying mechanism of this renoprotective effect may be associated with glycemic control, lipid metabolism, and anti-oxidative and anti-inflammatory functions [69]. Al Za’abi et al. found that metformin can be a useful drug in attenuating the progression of adenine-induced CKD in both diabetic and non-diabetic rats [70]. Increasing numbers of studies are investigating whether metformin exerts its hypoglycemic effect through the modulation of microbiome in the diabetic rat gut [71]. However, its underlying mechanism remains largely unclear, though it has been shown that DM leads to a higher Firmicutes/Bacteroidetes ratio in the gut microbiome that can be reverted by metformin [71,72].

**Figure 1:**
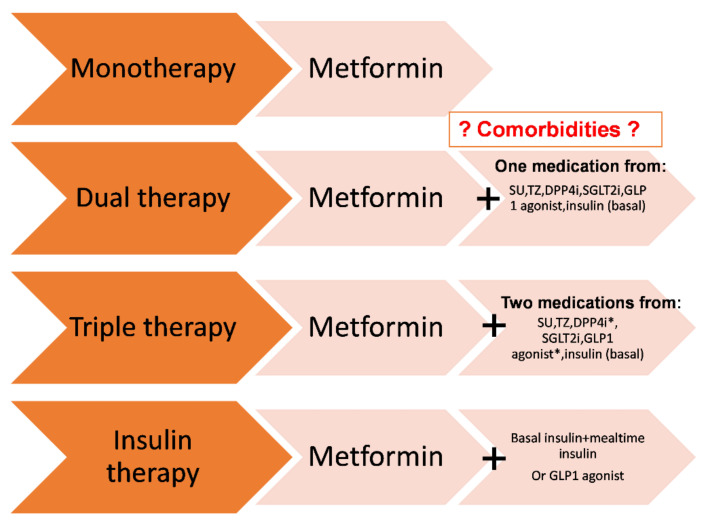
Summary of glucose-lowering medication in T2DM: monotherapy and combination of drugs. Adapted from the 2022 ADA Professional Practice Committee (PPC) based on the work of Davis and Busa et al. [75,76,77]. This strategy suggests a selection of therapy rather than sequential add-on, which may require the adjustment of ongoing therapies. Treatment should be individualized to comorbidities (such as heart failure, atherosclerotic cardiovascular disease, chronic kidney disease, and cardiovascular disease), patient-centered treatment factors, and management needs. DPP-4i, dipeptidyl peptidase 4 inhibitor; GLP-1 agonist, glucagon-like peptide 1 receptor agonist; SGLT2i, sodium–glucose cotransporter 2 inhibitor; SU, sulfonylurea; TZD, thiazolidinedione. * A treatment with DPP-4 inhibitors should be stopped when GLP-1 receptor agonists are used [78].

Among patients with T2DM who have established CVD or indicators of high risk, established kidney disease, or heart failure, glucagon-like peptide-1 receptor agonists (GLP-1 RAs) or a sodium–glucose cotransporter-2 inhibitor (SGLT2i) with demonstrated CVD benefits are recommended (Figure 1) [73].

There is increasing evidence to support the role of sodium–glucose cotransporter 2 inhibitor therapy in patients with CKD with or without T2DM. Individualized treatment with SGLT2i represents a promising therapeutic option for patients with diabetic and nondiabetic CKD to slow down disease progression [74]. SGLT2 inhibitors have a cardioprotective effect in the cardiovascular system. These have been recommended to treat heart failure with reduced ejection fraction (HFrEF), improving left ventricular ejection fraction and decreasing left ventricular end-diastolic diameter and pro-B-type natriuretic peptide level [75].

The first GLP-1 RA was approved by the FDA in 2005: exenatide BID injected twice daily. Later, other GLP-1 Ras, such as liraglutide and lixisenatide (injected once a day), and long-lasting drugs injected once weekly, such as exenatide, albiglutide, dulaglutide, and semaglutide, were developed [79]. The GLP-1 hormone is cleaved by DPP-4 (dipeptidyl peptidase-4) within minutes, so GLP-1 RAs were used for their resistance to DPP-4 in order to prolong their half-life and beneficial effects [76]. The majority of GLP-1 RAs are injectable glucose-lowering agents, with a low-risk of hypoglycemia via the stimulation of GLP-1 receptors leading to an increase in insulin secretion [77]. The first oral GLP-1 RA was Rybelsus^®^ (oral semaglutide), which was approved by the FDA in 2019 [80]. The effect of GLP-1 RAs is glucose-dependent, and they act as multi-target drugs on the (1) stimulation of pancreatic β-cell insulin production, (2) suppression of pancreatic α-cell glucagon secretion, and (3) suppression of hepatic glucagon synthesis with (4) the suppression of gastric emptying time, (5) increased satiety, and (6) increased insulin uptake at the peripheral tissues [81]. GLP-1 receptor agonists, except for lixisenatide, prevent the development and progression of coronary atherosclerosis, vasospasm of epicardial coronary arteries, and structural/functional changes in coronary microvasculature [82].

DPP-4 inhibitors (DPP-4i) also act via the incretin (intestinal secretion of insulin) effect through facilitating the glucose-dependent insulin secretion of pancreatic β-cells via prolonging the stability of the glucagon-like peptide-1 (GLP-1) and glucose-dependent insulinotropic polypeptide (GIP) [83]. DPP-4i are oral drugs administered once a day, though vildagliptin is administered twice a day [83]. The first DPP-4i was sitagliptin, which was approved by the FDA in 2006; others developed later include saxagliptin, vildagliptin, alogliptin, and linagliptin [84]. Our group showed that the DPP-4 inhibitor sitagliptin has a pleiotropic secondary cardioprotective effect and protects against ischemia-reperfusion injury through the modulation of NOS system and transient receptor potential (TRP) channels [85]. In agreement with Murase et al., the inhibition of DPP-4 enzyme improved survival after myocardial infarction in T2DM by modifying autophagy in the non-infarcted region of the heart [86]. Based on the above-mentioned studies, it is clear that DPP4 inhibitors have other beneficial effects that are not of a metabolic nature such as a cardioprotective effects, especially via decreasing systolic blood pressure independently of glucose-lowering effects. This is valuable because the prevalence of hypertension in T2DM patients is estimated to be twice higher than that of healthy individuals [87].

SGLT2i are oral antidiabetics that act via blocking glucose reabsorption in the proximal tubule of the nephron, leading to glucosuria irrespective of the insulin level [88]. The first SGLT2i, dapagliflozin, was approved by the European Medicines Agency (EMA) in 2013, and the others are canagliflozin, empagliflozin, tofogliflozin, and ipragliflozin (approved in Japan and Russia) [89]. It has been significantly verified by clinical studies that SGLT2i reduce the risk of a series of cardiovascular or renal complications such as atherosclerotic CVD, myocardial infarction, and CKD [90]. T2DM-induced sterile inflammation, endothelial dysfunction, and oxidative stress lead to vascular injury. SGLT2i, especially empagliflozin, revise glucotoxicity via glucosuria and significantly improve cardiovascular mortality in T2DM [91]. The same research team showed that there is an inverse correlation between endothelial function and serum HbA1c. Moreover, phagocytic leukocytes and C-reactive protein (CRP) were positively correlated with HbA1c. The viability of hyperglycemic endothelial cells was pleiotropically improved by SGLT2i [92]. Apart from their main pharmacological effect in DM, it has been found that SGLT2i may have novel therapeutic applications for diabetes, cardiovascular diseases, nephropathies, liver diseases, neural disorders, and cancer based on their antioxidant properties and unique perspective [93]. 

The early introduction of insulin should be considered if there is evidence of ongoing catabolism (weight loss), if symptoms of hyperglycemia are present, or when HbA1c or blood glucose levels are very high (HbA1c > 10% (86 mmol/mol) and blood glucose ≥16.7 mmol/L (300 mg/dL)) [94,95]. As T2DM progresses, most patients require treatment with basal insulin in combination with another agent to achieve recommended glycemic targets [96]. The ADA’s Standards of Medical Care in Diabetes recommend either starting an initial total daily dose of 10 units insulin or using a weight-based total daily dose from 0.1 to 0.2 units/kg. As a rule, the basal insulin dose may be increased by 2 units for every 20 mg/dL that the patient’s average fasting blood glucose level is over the recommended fasting blood-glucose level (<130 mg/dL per ADA guidelines) [73]. Despite the availability of the series of conventional therapies, in many cases, patients do not respond well to the given drug and undesirable side effects can even occur. Therefore, almost 1200 plants, the sources of natural products known as ethnomedicinal agents with reported anti-diabetic properties, are at the forefront of intensive research [97]. Among other molecules, many flavonoids possess anti-inflammatory effects in parallel with their ability to improve glucose metabolism [98]. Flavonoids, such as genistein, kaempferol, pectolinarin, and quercetin, have a profound anti-inflammatory effects with the stimulation of glycogen synthase [98,99]. In an animal model of T2DM, rutin decreased serum glucose concentration and inhibited protein-tyrosine-phosphatase 1B, a negative regulator of the insulin pathway [100]. A recent review summarized the wide repertoire and mechanisms of action of antidiabetic flavonoids [101]. Terpenoids are also reported as anti-diabetic compounds with benefits in the treatment of T2DM by normalizing blood glucose levels and advantages in the management of the retinopathy, nephropathy, neuropathy, and impaired wound healing [102]. Abscisic acid, a terpenoid phytohormone and an endogenous hormone in humans, can facilitate the release of insulin from β-pancreatic cells via the induction of GLP-1 [103].

## 7. Clinical Complications of T2DM

Severe T2DM may cause damage to complex organs such as the kidneys, eyes, and heart including vascular vessels [14]. Early-onset T2DM is associated with a greater lifetime risk of diabetes-associated complications than T1DM or late-onset T2DM [27,104]. Loss of hearing and problems with fertility are frequent complications with early-onset T2DM [27]. Diabetes mellitus (DM) is a chronic metabolic disorder associated with persistent hyperglycemia (>7 mmol/L (126 mg/dL in blood) [105]. Several factors can contribute to it: impaired insulin secretion, resistance to peripheral actions of insulin, or both. The progression of metabolic stress of hyperglycemia with the activation of Toll-like receptors, induction of endoplasmic reticulum stress, and activation of inflammasome may fuel chronic inflammation, thus augmenting pancreatic β-cell dysfunction and finally worsening patient conditions in T2DM [106]. Chronic and untreated hyperglycemia in synergy with other metabolic diseases in patients with diabetes mellitus can lead to the development of disabling and life-threatening health complications, most prominent of which are microvascular (retinopathy, nephropathy, and neuropathy) and macrovascular complications, as well as a 2-to-4-fold increased risk of cardiovascular disease. It has long been known that hyperglycemia-mediated pathways such as the polyol pathway, hexosamine biosynthetic pathway, advanced glycation end products (AGEs), and protein kinase C may harm cardiac endothelial cells [107]. While the intentions of DM treatment are to normalize the hyperglycemia and reduce the amount of HbA1c to a normal level, therapies of other metabolic disorders that are mainly associated with diabetes, such as dyslipidemia, hypertension, hypercoagulability, obesity, and insulin resistance, have also been major focuses of therapy and patient management [107].

Complications of diabetes are broadly divided into microvascular and macrovascular types. Microvascular complications include neuropathy, nephropathy, and retinopathy, while macrovascular complications consist of CVD, stroke, and peripheral artery disease (PAD) [11]. Diabetic foot syndrome has been defined as the presence of foot ulcer associated with neuropathy, PAD, and infection, and it is a major cause of lower limb amputation [108]. Finally, there are other complications of diabetes that cannot be included in the two aforementioned categories such as dental disease, reduced resistance to infections, and birth complications among women with GDM. CVD is one of the deadliest complications of T2DM, and patients with T2DM have two times the risk to develop CVD than those without T2DM [48,109]. A recent review summarized the risk of CVD in T2DM, shedding light on the idea that both BNP and pro-BNP may serve as predictive biomarkers of heart failure and CV mortality [110]. The higher risk for death of T2DM patients increases with the presence of CVD (hypertension), younger age (age <25 years), severe renal failure, fatty liver, hyperlipidemia, microalbuminuria, and worse glycemic control [11,111]. In a cross-sectional study including 1156 patients, the one-year mortality was found to be higher in T2DM patients with severe hypoglycemia than those without [112]. 

Obesity is one of the main modifiable risk factor leading to T2DM [113]. Additionally, obesity has frequently interconnected with high-grade systemic inflammation, thus promoting devastating immune activation in T2DM [114]. Factors released by the adipose tissue such as pro-inflammatory cytokines (tumor necrosis factor-α (TNF-α), interleukin-6 (IL-6), and IL-1b), non-esterified fatty acids, glycerol, and hormones may underly insulin resistance [115]. The disbalance of the immune system, the endothelial activation of monocytes, and macrophages in the adipose tissue may also release TNF-α and IL-6, thus exacerbating inflammation [116,117]. A meta-analysis of 20 clinical trials with 1065 T2DM patients versus 1103 healthy controls showed a correlation with monocyte activation and CVD risk in T2DM [116]. Obesity management with special diet, exercises resulting in at least 15% weight loss, and longer sleep may have great disease-modifying effect on T2DM [118,119]. The triglyceride–glucose (TyG) index is a potentially useful marker for predicting T2DM and has been reported to be associated with CVD risk. A higher TyG index value is associated with the presence of retinopathy and nephropathy in individuals with diabetes and could be used for monitoring metabolic status in clinical settings [120]. The mechanism of action of TyG in T2DM pathology is not evident, but it has been suggested that high blood glucose levels raise the level of reactive oxygen species (ROS) that mediate beta cell injury [121]. Higher TyG index values have shown clear associations between BMI and the development of T2DM [121,122].

Almost 10% of deaths caused by T2DM are referable as diabetic kidney disease (DKD) with renal failure [123]. Albuminuria and reduced eGFR are risk factors for end-stage kidney disease and CVD, as well as death [124]. One of the first clinical signs of such microvascular damage in diabetes is microalbuminuria [125]. Biomarkers predicting the progression of nephropathy in T2DM patients are plasma asymmetric dimethylarginine, serum interleukin-18 and urinary ceruloplasmin, immunoglobulin G, and transferrin [126]. 

The development of cancer was shown to have a positive correlation with T2DM in the case of colorectal, lung, esophagus, thyroid, bladder, hepatocellular, gallbladder, breast, endometrial, pancreatic and liver cancers [127,128,129]. Possible causes linking T2DM to increased cancer prevalence are diverse factors such as aberrant endocrine status, obesity, chronic inflammation, hyperglycemia with increased insulin level, and additional sedentary lifestyle factors [130]. Metformin was shown to reduce the risk of cancer development in T2DM via several indirect mechanisms: (1) reducing circulatory androgens, (2) inducing hepatic adenosine monophosphate kinase phosphorylation, (3) lowering blood glucose and gluconeogenesis, (4) reducing insulin level, and (5) exerting anti-inflammatory effects [130,131]. Metformin was also reported as an adjuvant that could increase the complete pathological response rate of HER2-positive breast cancer patients bearing the rs11212617 single-nucleotide polymorphism (SNP) located near the ataxia telangiectasia mutated (ATM) gene [132]. The association of T2DM and breast cancer was shown by the hypoxia-independent stabilization of HIF-1α via the insulin–PI3K–AKT, MAPK/ERK, IL-1, and NF-κB pathways. Subsequent HIF-1α-mediated events and the induction of glucose transporter GLUT1, glycogen synthase kinase, E-cadherin, and matrix metalloproteinases lead to epithelial–mesenchymal transition, the “entrance-hall” of cancer [133]. The risks and factors involved in cancer development in T2DM patients have been recently reviewed elsewhere [127,134]. 

## 8. Pathophysiology of T2DM and COVID-19

Hyperglycemia initiates a pathobiochemical cascade that results in increased mortality in SARS-CoV-2-infected diabetic patients [135,136]. The underlying molecular mechanisms are responsible for the worsening of both metabolic and hemodynamic conditions. A chronic glucose level leads to the hyperglycosylation of the ACE2 receptor and increased viral cell proliferation [137]. It has long been known that ACE2 is responsible for the conversion of angiotensin I into angiotensin II during the physiological state, and it has been identified as the receptor for SARS-CoV-2 viral entry into cells. ACE2, which directly interacts with the spike glycoprotein [138], is expressed in many cell types and is also present in epithelium of the lung at a high density. It has been shown that ACE2 is highly expressed in patients with hypertension, diabetes and coronary heart disease, thus leading to higher viral entry during SARS-CoV-2 infection. It is also well-known that T2DM is associated with both macrovascular and microvascular complications that lead to multiorgan failure, which worsens the outcome of COVID-19 in diabetic patients and increases mortality rates. The dysregulation of glucose metabolism and insulin resistance contribute to vasculopathy in both large and small vessels through various mechanisms [139]. Diabetes, mainly T2DM, is featured by chronic systemic inflammation and insulin resistance, which can result in endothelial dysfunction, oxidative damage, changes in the mitochondrial expression of superoxides, the increased formation of AGEs, and the activation of the receptors for advanced glycation end products (RAGE). The AGE–RAGE axis increases the progression of atherosclerotic legion formation in the arteries and thus accelerates vascular-damage-related conditions called diabetic vasculopathies [140]. However, the direct relationship between T2DM and COVID-19 remains complex; it is well-known that chronic hyperglycemia induces a dysregulated immune response in innate and adaptive immunity, including abnormal cytokine responses, the inhibition of leukocyte recruitment, the attenuation of macrophage and other leukocyte activity in eliminating pathogens, and defects in pathogen recognition and neutrophil functions [141]. Several other immune mechanisms, such as the decreased production of interleukins in response to an infection, reduced chemotaxis and phagocytic activity, and the immobilization of polymorphonuclear leukocytes, are affected in obesity. IFN-gamma released after virus infection downregulates the insulin-receptor expression of skeletal muscle, and viral infection enhances the progression of T2DM in obesity, thus worsening hyperglycemia [142]. Patients with T2DM tend to develop more severe forms of SARS-CoV-2 infection and have significant increases in acute phase proteins and inflammatory markers compared to non-diabetics. This may also enhance tissue tropism and viral penetration into the cells, leading to increased virulence, pathogenicity, and susceptibility to severe infections [143]. In patients suffering from COVID-19, DM was found to be the third most common comorbidity, with a 33.8% prevalence, after hypertension and obesity [144]. Several mechanisms have been suggested as an underlying additional explanation for the more severe course of COVID-19 in patients with diabetes. Behind the impaired immune system, hyperglycemia and hyperinsulinemia diabetes are also associated with a hypercoagulable state. The metabolic disturbances associated with oxidative stress and impaired immunity may accelerate the occurrence of thrombotic and ischemic events.

Patients with diabetes generally have an increased risk of thrombosis, which, in the case of COVID-19, can add to a high risk of death. Endothelial cell dysfunction plays a key role in the initiation and precipitation of thrombosis. The initiation of this process when the nitric oxide synthesis is decreased in endothelial cells via several mechanisms including the activation of NF-κB and protein kinase C (PKC) leads to the impairment of vasodilation, the expression of adhesion molecules, and the worsening of vascular inflammation. This results in increased platelet activation and a prothrombotic/hypofibrinolytic environment that facilitates thromboembolic events [145]. It is still unclear whether the dysregulation of glucose metabolism, the severe COVID-19 effects, or the SARS-CoV-2 infection itself is responsible for the worsening of carbohydrate metabolism in diabetic patients. The associations between glycemic control and short- to long-term outcomes were examined in a multi-center prospective cohort study including 574 COVID-19 patients; a one year follow up showed that the glycemic control was significantly associated with short-term outcomes in COVID-19 patients with T2DM and decreased the risk of respiratory sequelae [146]. In a German study of about 8.8 million people, 35,865 were infected by COVID-19, and 15.8 per 1000 person-years versus 12.3 per 1000 person-years of these patients developed T2DM versus other upper respiratory infections, respectively [147]. The results of that study suggest that SARS-CoV-2 infection may also increase the risk of developing T2DM. Future studies will answer the questions of whether SARS-CoV-2 really can induce T1DM, T2DM, or even a new type of diabetes. Long-term follow up studies are needed to evaluate whether the virus has a diabetogenic impact on patients with a higher risk for DM or it can stimulate a new type of DM [147].

## 9. Prognosis of T2DM Patients with COVID-19

The novel coronavirus, SARS-CoV-2, infected more than 500 million and caused the coronavirus disease 2019 (COVID-19) with the death of more than 6 million people worldwide by July 2022 (online COVID-19 Data Repository at Johns Hopkins University). Metabolic diseases such as DM are associated with an increased risk of a severe COVID-19 illness and death because of their associated hypercoagulation state and uncontrolled inflammation [148], although it seems that T1DM patients have higher risk than T2DM patients. Epidemiological studies have shown that hospitalization with diabetes and SARS-CoV-2 infection represents as a comorbidity with poor outcome during hospital stay [149]. A recent study reported that 15% of T2DM patients died from COVID-19, with the poor prognoses for those of elder age and elevated glucose and serum amyloid A levels [150]. In a Swedish study of 385,021 T2DM patients, an elevated glycemic hemoglobin level was shown as a bad prognostic factor and the risks for hospitalization, admission to intensive care, and fatal outcome of T2DM patients with COVID-19 were twice those of a control group [151]. On the contrary, the control of the glycemic index was significantly associated with less mortality and hospital stay in an analysis of 574 T2DM patients with COVID-19 in China [146]. Upon SARS-CoV-2 infection, T2DM patients had adjusted odds ratios (ORs): 3.36 for hospitalization, 3.42 for disease severity, and 2.02 for death [152,153]. In a national cohort study of 19,256 subjects in England conducted between March and July 2020, 18.3% of hospitalized COVID-19 patients also had T2DM [136]. In a Spanish study, 30.05% versus 19.57% was the ratio of deceased versus surviving diabetes patients, respectively [154]. Taken together, the risks of SARS-CoV-2 infection in patients with T2DM are well-documented and urge the prioritization for vaccination [148,155]. In an Italian study of 277 T2DM subjects (83.4% received an mRNA-based vaccine of mRNA-BNT162b2 or mRNA-1273 and 16.6% received a viral vector-based vaccine of ChAdOx1-S), the neutralizing antibody level and the number of SARS-CoV-2-reactive T-cells (CD4+/TNF-α+, CD4+/IL-2+, CD4+/IFN-γ+) were higher in patients with good glycemic control (HbA1c < 7%) at 52 days after the second vaccine [156]. In a retrospective clinical study of 1356 T2DM patients hospitalized with COVID-19, it was shown that the metformin-treated group showed less mortality and shorter stays in hospital, probably due to the anti-inflammatory effect of metformin [157]. However, T2DM therapy should be designed in accordance with local guidelines while taking personal parameters and comorbidities into account; therefore, current therapeutic regimens for the management of T2DM are not discussed here. Recent review articles about the management of COVID-19 in patients with T2DM have been published elsewhere [158,159,160].

Angiotensin-converting enzyme 2 (ACE2) is one of the best-characterized proteolytic enzymes and a functional receptor on cell surfaces through which SARS-CoV-2 enters the cells. ACE2 is abundantly found in the lung alveolar epithelial cells, lung vascular endothelial cells, heart, kidneys, and pancreas [161]. However, controversial results have been found regarding the expression profile of the ACE2 protease enzyme and receptor. Some studies have suggested that it is more preferably expressed in the exocrine duct cells than in the islets, whereas other studies have shown that ACE2 is expressed in beta-cells; moreover, ACE2 was detected in the microvasculature of both the exocrine and endocrine pancreas. These discrepancies were clarified by Stellenbock et al., who examined the expression of ACE2 in pancreatic autopsy tissues from eleven patients that died of COVID-19. They found that the pancreata were infiltrated with CD45-positive immune cells and that mainly beta cells were infected by SARS-CoV-2 virus. They speculated that other receptors/entry-points may be involved in facilitating the uptake of SARS-CoV-2 into beta-cells because the ACE2 positivity of beta cells was only detected in some the human subjects [162]. 

Among other risk factors for COVID-19-related death, DM has been shown one of the main predictors of the SARS-CoV-2 infection-associated mortality rate. Therefore, we reviewed the most relevant pathobiochemical aspects, summarized the known molecular background of SARS-CoV-2-induced pathomechanical abnormalities, and dissected the current prognosis of COVID-19 patients in T2DM.

## 10. The Potential Role of Multi-Omics and Single Cell-Based Technologies in the Current Research of T2DM

In the last decade, the “multi-omics” approaches reached a breakthrough in understanding the pathomechanism and clinical complications of T2DM. Different next-generation sequencing (NGS) and mass-spectrometry-based genomics and metagenomic approaches have emerged and are used to identify possible disease-associated diagnostic or therapeutic targets from affected tissues and blood based on specific gene expression changes including those of diabetes (Table 4) [163,164,165,166,167]. The NGS analysis of 16S rRNA genes showed that comorbidity of T2DM with HIV led to a lower microbiome diversity, which was negatively impacted by smoking and normalized by metformin treatment [168]. In the study of Tong et al., the sequencing of 16S rRNA by NGS revealed that metformin treatment increased the proliferation of *Blautia* spp. in the gut in correlation with the normalization of hyperglycemia and hyperlipidemia [169]. An analysis of 40 single-nucleotide polymorphisms (SNPs) in 40 genes of 503 T2DM patients vs. 580 healthy controls on a Sequenom platform identified SNPs in the CAT, FTO and UCP1 genes associated with the retinopathy and nephropathy complications of T2DM [170]. Although early GWAS studies identified approximately 75 genetic loci associated with the development of T2DM, recent multi-ancestry genetic studies found more than 500 risk loci, and the heritability of T2DM via these genes has been shown in only 10–15% of cases, so it is more likely that lifestyle and environmental factors have a significant additional effect that contributes to the manifestation of T2DM [15,16,17,171].

Many epigenetic studies, including the investigation of DNA methylation patterns and accessible chromatin profiles in different tissues, have also contributed to our current knowledge of T2DM [172,173]. One of the most extensive epigenome-wide association studies (EWAS) revealed the CpGs methylation pattern of 52 genes in the blood of European T2DM subjects with the Illumina 450 K methylation array and identified five genes with altered CpG methylation patterns—ABCG1, LOXL2, TXNIP, SLC1A5 and SREBF1—that were significantly associated with the disease [174]. Using “Assay for Transposase-Accessible Chromatin with high throughput sequencing” (ATAC-seq method), Ackermann et al. determined the human pancreatic alpha or beta cell-specific open chromatin landscape and found that alpha or beta cell-specific ATAC-seq peaks overlapped with known binding motifs for various transcription factors, including alpha cell-specific ISL1 and MAFB or beta cell-specific SMAD2, as well as previously identified T2DM-risk-associated SNPs [175]. Greenwald and colleagues combined a high-throughput chromosome conformation capture technique (Hi-C) assay-based high-resolution map of islet chromatin loops with the ATAC-seq and publicly available chromatin immunoprecipitation sequencing (ChIP-seq) data-defined enhancers. They identified thousands of pancreatic islet-specific enhancer–target gene pairs. The T2DM-risk-linked SNPs were significantly enriched at the active enhancers of the protein transport and secretion pathway-associated genes. In the case of the IGF2BP2 gene, the identified T2DM-specific SNP could attenuate both islet enhancer activity and IGF2BP2 expression, and the islet-specific conditional deficiency of Igf2bp2 gene led to impaired glucose-induced insulin secretion in mice [176].

Recently, several research groups started to study the development and progression of T2DM in human patients by applying state-of-the-art single-cell RNA-sequencing (scRNA-seq) and single-sell ATAC-sequencing (scATAC-seq) methods focusing on the pathological changes in pancreatic islets. Lawlor and colleagues investigated the cellular heterogeneity in nondiabetic and T2DM human islet samples, and they were able to detect T2DM-specific gene expression signatures in alpha, beta, and delta cells using scRNA-seq that remained invisible in paired whole-islet analyses [177]. Additionally, scRNA-seq and complex computational tools revealed an altered regulatory network in the pancreas of T2DM patients with disease-related transcriptomic changes, showing increased PageRank centrality in 162 genes. After analyzing five centralities driving the regulatory changes in diabetes, they found six markers with increased levels (OTUD7B, PPRC1, ARRB2, C17orf96, NME2, and E2F1) and four markers with decreased centrality (FBXW7, CXCL8, FHL1, and CELF4) [178]. By applying scATAC-seq and deep learning approaches, Rai et al. found that T2DM-associated SNPs were significantly enriched in beta cell-specific and common islet-specific open chromatin but not in alpha or delta cell-specific open chromatin signatures [179]. Marques et al. performed a meta-analysis of the scRNA-seq data of human α- and β-cells of T2DM patients and identified disease-associated genes responsible for energy metabolism, immune homeostasis, autophagy, and especially nuclear factor erythroid 2-related factor 2 (NFE2L2) in β-cell maturation and dysfunction [180].

The manifestation of T2DM in Asian Indians is more frequent, even in the case of normal BMI, a situation known as the “thin fat” phenotype in which the peripheral fat is thin but the visceral fat accumulates [181]. Microarray data of T2DM-derived peripheral fat of Asian Indians were sued to highlight the top 20 differentially expressed genes (DEGs) and pathways associated with adiposopathy in T2DM [182]. Using the whole transcriptome RNAseq, the same group further investigated the peripheral subcutaneous adipose tissue of Asian Indians and found altered lipid, glucose, and protein metabolisms; adipogenesis defects; and inflammation associated with T2DM [183]. Using the AGENA MassARRAYiPLEX™ platform, Irgam et al. recently identified seven significant SNPs (s2241766-G (ADIPOQ), rs6494730-T (FEM1B), rs1799817-A, rs2059806-T (INSR), rs11745088-C (FST), rs9939609-A, and rs9940128-A (FTO)) associated with T2DM in a southern Asian Indian population of 500 cases [184].

Besides genomics studies, multiplex proteomic investigations have revealed markers associated with disease severity or complications in T2DM. Using the Milliplex Luminex assay, Barchetta et al. showed that blood levels of osteopontin and osteoprotegerin were significantly higher in 83 T2DM patients versus 71 healthy controls and that these proteins were positively correlated with higher systolic blood pressure [185]. Using the same multiplex Luminex technology, Colombo et al. showed that the elevated serum concentrations of kidney injury molecule 1 (KIM-1) and β2-microglobulin (B2M) were correlated with renal failure and a decreased glomerular filtration rate in T2DM [186]. The study of Heinzel also based on the Luminex quantitation of plasma biomarkers identified KIM-1 among 12 proteins of 17 measured markers that predicted declines in the glomerular filtration rate [187]. Although T2DM is not autoimmune-mediated, using single-cell imaging mass cytometry, Wu et al. showed increased percentages of HLA-DR+ macrophages and HLA-DR+ CD8+ T-cells in the islets of the pancreata of T2DM patients, thus suggesting their role in local inflammation [188]. Novel experimental models can also be used to understand better the pathomechanisms of different diabetic syndromes. Our group was to first to optimize a special three-dimensional organoid, the Real Architecture For 3D Tissue (RAFT™) culture system, for the ex vivo maintenance of functional murine pancreatic islets [189].

A lipidomics study of 250 T2DM patients and 639 non-cases showed that the plasma lipid profiles of elevated TAGs (triacylglycerols), DAGs (diacylglycerols), and PEs (phosphatidylethanolamines) with a high risk of T2DM and lipid constituents such as LPs (lysophospholipids), PC–PLs (phosphatidylcholine–plasmalogens), SMs (sphingomyelins), and CEs (cholesterol esters) were associated with lower risks of T2DM [190]. In a Finnish lipidomics study analyzing 277 plasma lipids with ultra-performance liquid chromatography coupled to time-of-flight mass spectrometry of 955 subjects with a 5-year follow-up also found increases in TAGs and DAGs and decreases in PC–PLs associated with risk of T2DM [191]. Taken together, the disbalance of fatty acids (FAs) may not only be considered as a consequence of altered metabolism; rather, FAs may be involved in the translocation of glucose transporters and influence insulin receptor binding as causative agents in the development of T2DM [192].

**Table 4:** Recent multi-omics approaches that revealed T2DM-associated factors.

Omics/Field	Measures	Results	Assay	References
Genomics	16S rRNA on microbiome analysis	Smoking and/or HIV lowers microbiome diversity in T2DM	NGS	[168]
Genomics	16S rRNA on microbiome analysis	Metformin helps to normalize microbiome with the support of *Blautia* spp.	NGS	[169]
Genomics	Analysis of SNPs	SNPs in the CAT, FTO and UCP1 genes associated with retinopathy and nephropathy	Sequenom platform	[170]
Genomics	Genome sequencing	Heritability of T2DM is approximately 10–15%	GWAS	[15,16,17,171]
Epigenomics	CpGs methylation pattern	CpG methylation of ABCG1, LOXL2, TXNIP, SLC1A5 and SREBF1 is associated with T2DM	EWAS, Illumina 450K methylation array	[174]
Epigenomics	Alpha or beta cell-specific open chromatin landscape	Alpha cell-specific ATAC-seq peaks: ISL1 and MAFB; beta cell-specific: SMAD2	ATAC-seq	[175]
Epigenomics Genomics	Open chromatin regions/SNPs	Thousands of pancreatic islet-specific enhancer–target gene pairs	Hi-C, ATAC-seq, ChIP-seq	[176]
Transcriptomics	Gene expression	T2DM-specific gene expression signatures in alpha, beta and delta cells	scRNA-seq	[177]
Transcriptomics	Gene expression, regulatory networks	Increased OTUD7B, PPRC1, ARRB2, C17orf96, NME2, and E2F1 or four markers with decreased PageRank centrality (FBXW7, CXCL8, FHL1, and CELF4)	scRNA-seq	[178]
Epigenomics Genomics	scRNA-seq and deep learning approaches	T2DM-associated SNPs were significantly enriched in beta cell-specific and common islet-specific open chromatin	scRNA-seq and deep learning approaches	[179]
Transcriptomics	Gene expression, pathway analysis	T2DM-associated genes responsible for energy metabolism, immune homeostasis, and autophagy	Meta-analysis of scRNA-seq data	[180]
Transcriptomics	Whole transcriptome analysis	Top DEGs in peripheral fat of Asian Indians associated with T2DM: *HOXB3*, *RSPO3*, *HOXA5*, *GREM1*, *ORMDL1*, *C7*, *TRIM23*, *CLDN11*, *ABCA10*, *ETV5*, *TRIM2*, *TP53INP1*, *ST6GAL1*, *THBS2*, *ERAP1*, *OGT*, *RARRES1*, *CTDSPL* and *TBCC*	Affymetrix GeneChip PrimeView Human Gene Expression Array	[182]
Transcriptomics	Whole transcriptome analysis	Altered lipid, glucose, and protein metabolism; adipogenesis defect; and inflammation in peripheral fat of Asian Indians associated with T2DM	Bulk RNAseq	[183]
Genomics	Analysis of SNPs	s2241766-G (ADIPOQ), rs6494730-T (FEM1B), rs1799817-A, rs2059806-T (INSR), rs11745088-C (FST), rs9939609-A, and rs9940128-A (FTO) were associated with T2DM in southern Asian Indians	AGENA MassARRAYiPLEX™ platform	[184]
Proteomics	Protein concentrations	Osteopontin and osteoprotegerin are elevated in T2DM	Milliplex Luminex assay	[185]
Proteomics	Protein concentrations	High KIM-1 and β2-B2M are associated with renal failure	Luminex Multiplex ELISA Luminex assay	[186]
Proteomics	Protein concentration	High KIM-1 is associated with low GFR	Multiplex Luminex Panel	[187]
Proteomics	Immune cell infiltration	High HLA-DR+ macrophages and HLA-DR+ CD8+ T-cells in the islets of pancreata of T2DM patients	Single-cell imaging mass cytometry	[188]
Lipidomics	Lipid composition	High TAGs, DAGs, PEs: high risk for T2DM High LPs, PC–PLs, SMs, CEs: low risk for T2DM	Mass spectrometry (MS)	[190]
Lipidomics	Lipid composition	High TAGs, DAGs and Low PC–PLs: high risk for T2DM	Ultra-performance liquid chromatography and MS	[191]

Taken together, the above-discussed studies illustrate the relevance of multi-omics and single cell-based technologies in the study of T2DM pathomechanisms. However, many questions remain unanswered, including (i) which islet-specific enhancers/open chromatin regions are associated with the different therapeutic responsiveness levels, (ii) how non-pharmacological and pharmacological treatments can modulate the cellular heterogeneity in the pancreas, and (iii) which gene expression and epigenetic signatures or plasma biomarkers may be helpful to predict the therapeutic responsiveness in T2DM patients. The limitations of genome-based or transcriptome-based investigations, such as lack of functional tests, a lack of functional evaluation of metabolic traits, and protein circuits in cell-to-cell communication, should be considered. Proteomics and lipidomics approaches also share limitations with the aforementioned technologies, with questions of assay sensitivity, sample preparation, and throughput, among others. No single omics technology can identify and quantify the T2DM-related factors responsible for both disease heredity, manifestation, and severity or serve as a therapeutic target or prognostic/diagnostic marker. Rather, the combination of the presented technologies may accelerate the understanding of the molecular pathomechanism of T2DM and finally ameliorate patient health. Overall, individuals’ responsible lifestyle choices, lower calorie intakes, regular physical exercises, and SARS-CoV-2 vaccination may reduce the risk of the development or increased severity of existing T2DM.

## Figures and Tables

**Table 1:** Classification of main types of diabetes mellitus [2,3,4,5,7].

**Main types of diabetes mellitus**
Type 1 diabetes mellitus Type 2 diabetes mellitus Hybrid forms of diabetes Slowly evolving immune-mediated diabetes of adults Ketosis prone type 2 diabetes
**Other specific types**
Monogenic defects of β-cell function Monogenic defects in insulin action Diseases of the exocrine pancreas Endocrine disorders Drug- or chemical-induced Infection-related diabetes Uncommon specific forms of immune-mediated diabetes Other genetic syndromes sometimes associated with diabetes
**Unclassified diabetes**
Hyperglycemia first detected during pregnancy Diabetes mellitus in pregnancy Gestational diabetes mellitus

**Table 2:** The following criteria are used to establish a diagnosis of diabetes (Reference: [7]).

Fasting plasma glucose (8 h no food intake) level ≥126 mg/dL (7.0 mmol/L)
75 g OGTT 2 h value ≥ 200 mg/dL (11.1 mmol/L); OGTT: glucose load containing the equivalent of 75 g anhydrous glucose dissolved in water.
Hemoglobin A1c ≥ 6.5%
Random plasma glucose ≥200 mg/dL (11.1 mmol/L), sometimes appears as a hyperglycemic crisis
Clinical symptoms of diabetes (e.g., thirst, polydipsia, polyuria, weight loss, and dry mouth)

**Table 3:** Oral hypoglycemic medications approved by FDA indications [65,66].

Pharmacological Group	Drug	Biochemical Key Factor for Mechanism of Action	Mechanism of Action
Sulfonylureas (SU)	glipizide glyburide gliclazide glimepiride	K-ATP channels of beta cells	Close ATP-dependent potassium channels that depolarize the beta cells, opening calcium channels and causing insulin release
Meglitinides	repaglinide nateglinide	K-ATP channels of beta cells	Same as SU
Biguanides	metformin	Increase hepatic AMP-activated protein kinase activity	Reduce hepatic gluconeogenesis and lipogenesis, stimulate fatty acid oxidation, and increase insulin-mediated uptake of glucose in muscles
Thiazolidinediones (TZD)	rosiglitazone pioglitazone	Activate peroxisome proliferator-activated receptor gamma (PPAR-γ)	Increase insulin sensitivity and stimulate fatty acid oxidation
α-Glucosidase inhibitors	acarbose miglitol voglibose	Inhibit alpha-glucosidase enzymes in the intestinal brush border cells	Inhibit polysaccharide reabsorption
GLP-1 Receptor Agonists	exenatide BID liraglutide lixisenatide exenatide albiglutide, dulaglutide semaglutide oral semaglutide (Rybelsus)	Stimulate GLP-1 receptors	Lead to the increase in insulin secretion
DPP-4 inhibitors	sitagliptin vildagliptin saxagliptin linagliptin alogliptin	Inhibit the enzyme dipeptidyl peptidase 4 (DPP-4)	Decrease glucagon release, thus increasing glucose-dependent insulin release
SGLT2 inhibitors	dapagliflozin canagliflozin empagliflozin tofogliflozin	Inhibit sodium–glucose cotransporter 2 (SGLT-2) in the proximal tubules of renal glomerulus	Inhibition of glucose reabsorption, resulting in glycosuria
Cycloset	bromocriptine	Dopamine (D2) receptor agonist	Resets the hypothalamic circadian rhythm and improves insulin resistance
